# Genetic Variants from Lipid-Related Pathways and Risk for Incident Myocardial Infarction

**DOI:** 10.1371/journal.pone.0060454

**Published:** 2013-03-29

**Authors:** Ci Song, Nancy L. Pedersen, Chandra A. Reynolds, Maria Sabater-Lleal, Stavroula Kanoni, Christina Willenborg, the CARDIoGRAMplusC4D Consortium, Ann-Christine Syvänen, Hugh Watkins, Anders Hamsten, Jonathan A. Prince, Erik Ingelsson

**Affiliations:** 1 Department of Medical Epidemiology and Biostatistics, Karolinska Institutet, Stockholm, Sweden; 2 University of California Riverside, Riverside, California, United States of America; 3 Atherosclerosis Research Unit, Department of Medicine Solna, Karolinska Institutet, Stockholm, Sweden; 4 Wellcome Trust Sanger Institute, Hinxton, United Kingdom; 5 Universität zu Lübeck, Medizinische Klinik II, Lübeck, Germany; 6 Deutsches Zentrum für Herz-Kreislauf-Forschung (DZHK), Lübeck, Germany; 7 Department of Medical Sciences, Uppsala University, Uppsala, Sweden; 8 Department of Cardiovascular Medicine, The Wellcome Trust Centre for Human Genetics, University of Oxford, Oxford, United Kingdom; 9 Department of Cardiovascular Medicine, University of Oxford, John Radcliffe Hospital, Headington, Oxford, United Kingdom; 10 Center for Molecular Medicine, Karolinska University Hospital, Stockholm, Sweden; 11 Department of Medical Sciences, Molecular Epidemiology and Science for Life Laboratory, Uppsala University, Uppsala, Sweden; Universite de Montreal, Canada

## Abstract

**Background:**

Circulating lipids levels, as well as several familial lipid metabolism disorders, are strongly associated with initiation and progression of atherosclerosis and incidence of myocardial infarction (MI).

**Objectives:**

We hypothesized that genetic variants associated with circulating lipid levels would also be associated with MI incidence, and have tested this in three independent samples.

**Setting and Subjects:**

Using age- and sex-adjusted additive genetic models, we analyzed 554 single nucleotide polymorphisms (SNPs) in 41 candidate gene regions proposed to be involved in lipid-related pathways potentially predisposing to incidence of MI in 2,602 participants of the Swedish Twin Register (STR; 57% women). All associations with nominal P<0.01 were further investigated in the Uppsala Longitudinal Study of Adult Men (ULSAM; N = 1,142).

**Results:**

In the present study, we report associations of lipid-related SNPs with incident MI in two community-based longitudinal studies with in silico replication in a meta-analysis of genome-wide association studies. Overall, there were 9 SNPs in STR with nominal P-value <0.01 that were successfully genotyped in ULSAM. rs4149313 located in ABCA1 was associated with MI incidence in both longitudinal study samples with nominal significance (hazard ratio, 1.36 and 1.40; P-value, 0.004 and 0.015 in STR and ULSAM, respectively). In silico replication supported the association of rs4149313 with coronary artery disease in an independent meta-analysis including 173,975 individuals of European descent from the CARDIoGRAMplusC4D consortium (odds ratio, 1.03; P-value, 0.048).

**Conclusions:**

rs4149313 is one of the few amino acid changing variants in ABCA1 known to associate with reduced cholesterol efflux. Our results are suggestive of a weak association between this variant and the development of atherosclerosis and MI.

## Background

Circulating lipids concentrations, such as serum low-density lipoprotein cholesterol (LDL) and high-density lipoprotein cholesterol (HDL) levels are routinely used biochemical measures in clinical practice [1], since they are related to the development of atherosclerotic plaques and subsequent myocardial infarction (MI) [2]. A recent GWAS meta-analysis has identified 95 loci associated with circulating lipid levels[3], and genetic polymorphisms modifying function or expression of these genes are likely to be associated with MI as well. Several Mendelian diseases have provided support for this hypothesis. For example, familial hypercholesterolemia caused by mutations in the low-density lipoprotein receptor gene (*LDLR*) can elevate LDL levels and increase MI risk in early middle age [4]. Therefore, investigating variants in genes from lipid-related pathways may promote a greater understanding of the pathological process of atherosclerosis and assist in the search for novel treatment targets for this condition and subsequent MI.

The variety of genetic associations for serum lipid traits provides an intriguing basis from which to test if these same loci also impact risk for MI. In this study, we therefore targeted genetic loci associated with serum lipids, and tested these for association with MI incidence and symptomatic coronary artery disease (CAD) in two independent longitudinal studies with *in silico* replication in two large datasets.

## Methods

### Study samples

Our discovery sample consisted of four sub-studies within the Swedish Twin Registry (STR) [5]. Briefly, STR contains data regarding health, health-related behaviors, physical activity, eating habits, and environmental stressors along with other information from Swedish national registries. In current study, we utilized four sub-studies within STR: Sex differences in health and aging (GENDER) [6], Individual differences among the oldest-old (OCTO-Twin) [7], The Swedish Adoption/Twin Study of Aging (SATSA) [8], and the Study of Dementia in Swedish Twins (HARMONY) [9]. All data collection in these four sub-studies followed a similar fashion with both questionnaires and blood sampling as decribed in the previous study [10]. In total, we included 2,602 individuals with DNA available (sample call rate>90%). We also collected information of circulating lipids levels, including total cholesterol, LDL, HDL, triglycerides, apolipoprotein AI and apolipoprotein B for these individuals. All the blood tests were taken after overnight fasting, except for individuals from OCTO-Twin; thus, lipid analyses were adjusted for fasting status.

As an independent replication sample, we used the Uppsala Longitudinal Study of Adult Men (ULSAM), a community-based longitudinal study of unrelated individuals[11]. All men born in 1920–1924 who were residents of Uppsala County were invited to a health examination. All men participated in the investigation at age 50[12], and they have been re-examined five times so far. The DNA was collected at the re-examination at age 71 (n = 1,142), constituting the baseline information for the present study. Information about circulating lipids levels was also collected at age 71 after overnight fasting.

All participants provided informed written consent, and the Ethics Committees of Karolinska Institutet and Uppsala University approved the individual study protocols.

### Follow-up and outcomes

We collected outcome data from the Swedish Patient Register and the Cause of Death Register by linkage using the personal identification numbers. In primary analyses, we used age as the time-scale, with start of follow-up at age 18. In sensitivity analyses, we re-analyzed our top findings using date of blood draw in the respective cohort as start of follow-up. This allowed us to address the potential issue of survival bias and left truncation due to incomplete outcome information in the earlier years of the registries. Study participants were censored at their first occasion of MI, at the date of death or on Dec 31, 2008.

The occurrence of MI was defined by International Classification of Diseases (ICD) codes: ICD-7 codes before 1968, 420.10, 420.17; ICD-8 code from 1968 to 1986, 410; ICD-9 code from 1987 to 1996, 410; and ICD-10 codes from 1997 to present, I21 and I22. Only the main diagnoses (i.e. principal cause of hospitalization or underlying cause of death, respectively) were included in the outcome definition to increase the validity of the outcome (positive predictive value of ∼95%).[13], [14]

### Genetic marker selection and genotyping

Candidate genes were selected based on their reported associations with serum LDL, HDL or triglycerides through a literature search in publications from 2003 to 2008. All known polymorphisms within or in LD with potential gene regions based on CEU samples of HapMap Data Release 22 were taken into consideration. We used Haploview 4.0[15] to identify LD blocks, and to tag SNPs. SNPs in a region about 20 kb upstream and 10 kb downstream of the examined genes were selected using the UCSC Genome Browser[16]. After selection, Illumina scores for all markers were calculated; those that did not satisfy the criteria for Illumina probe chemistry were then replaced with an SNP in perfect LD [r^2^ = 1] if available or other tagging SNPs.

Genotyping was performed using the Illumina GoldenGate assay system on Illumina BeadStation 500GX equipment at Uppsala University SNP Technology Platform[17]. Monomorphic SNPs, SNPs with a call rate less than 90%, and SNPs which failed Hardy-Weinberg equilibrium (exact P-value<1*10^−5^) were excluded. A total number of 554 SNPs from 41 gene regions were included in the primary analyses in STR. All genetic markers studied are listed in **Supplementary** **Table 1** **in File S1**.

**Table 1:** Clinical characteristics of the study samples at time of DNA collectionα.

Clinical characteristics	STR (n = 2,602)	ULSAM (n = 1,142)
Ageβ, years	75±10	71±1
Women, %	57	0
Systolic blood pressure, mmHg	154±25	146±19
Diastolic blood pressure, mmHg	84±12	84±9
Anti-hypertensive medication, %	36.9	34.6
Lipid medication, %	1.8	9.6
Pulse rate, beats/min	71±12	65±9
Body mass index, kg/m^2^	25.1±6.2	26.3±3.4
Waist/hip ratio	0.9±0.1	0.9±0.1
Total cholesterol, mmol/L	6.1±1.4	5.8±1.0
High-density lipoprotein cholesterol, mmol/L	1.4±0.4	1.3±0.4
Low-density lipoprotein cholesterol, mmol/L	3.8±1.1	3.9±0.9
Triglycerides, mmol/L	1.7±0.9	1.4±0.8
Current smokers, %	7.4	18.0
Diabetes, %	6.3	14.4

α Data are means ± standard deviations or percentages;

β Age here refers to the age when the DNA samples.

As an independent replication effort in ULSAM, we used a combination of available genotype data (*in silico* replication) and *de novo* genotyping when needed. For the *in silico* replication, we only considered SNPs in strong LD with our SNP from the primary analysis (r^2^>0.8 according to HapMap rel22 CEU panel). The *de novo* genotyping was performed at Uppsala University SNP Technology Platform with the same methods as described above.

### Statistical analyses

Baseline covariates were presented as means with standard deviations or percentages. We performed age- and sex-adjusted Cox proportional hazards regression with additive genetic models for association analyses between SNPs and first MI occurrence in STR as our primary analyses. We constructed null data sets through bootstrap re-sampling to account for multiple statistical testing. For each of the 554 SNPs, we ran the regression model 10,000 times and obtained an empirical probability value for each phenotype by comparing the minimum nominal probability value with the distribution of probability values from the null data sets.

All SNPs with nominal P-values for association with MI of less than 0.01 in our discovery analyses were brought forward to replication, and to additional analyses, such as associations with lipid levels. In additional analyses, we fitted Cox models with shared frailty to take the twin relatedness into account[18] and did sex-stratified analyses. Besides, we performed multilevel mixed-effects linear regression models adjusted by age, sex and fasting status to study associations of these SNPs with total cholesterol, LDL, HDL, triglycerides, apolipoprotein AI and apolipoprotein B in additive genetic models in STR. Then independent replication in ULSAM was performed for association of those SNPs with MI and with lipids using age-adjusted Cox regression and linear regression, respectively.

In a *post-hoc* power calculation, we had 51.8% statistical power to detect a hazard ratio of 1.36 in the discovery sample at an alpha level of 0.01 in our discovery sample. Two-tailed 95% confidence intervals and P-values were given. All results reported in the current study were nominal P-value without correcting multiple tests. All analyses above were performed using statistical software package STATA 10.1 (StataCorp, College Station, TX, USA).

### *In silico* replication in CARDIGRAMplusC4D

We performed *in silico* replication of the single variant (rs4149313) that showed evidence of association in both STR and ULSAM in a meta-analysis of 40 independent studies from the CARDIOGRAMplusC4D consortium. This effort combined GWAS and Metabochip studies of European and South Asian descent[19]. After excluding STR and ULSAM (as being part of our discovery and first replication stages), as well as three studies with individuals of South Asian descent, we included 173,975 individuals in this meta-analysis using fixed-effect models. All cases were identified as have symptomatic coronary artery disease (CAD), including MI, angina pectoris, history of percutaneous transluminal coronary angioplasty (PTCA) or coronary artery bypass graft surgery (CABG). Since rs4149313 was not available in the all studies of CARDIoGRAMplusC4D consortium, a proxy in perfect LD (rs4149311; r2 = 1.0) was used instead for these analyses. All analyses were done using statistical software package STATA 11.2 (StataCorp, College Station, TX, USA).

## Results

### Baseline characteristics of present study

The clinical characteristics at the time of DNA collection of the study samples are presented in **Table 1**. In STR, 397 individuals developed a first MI during their entire life course. The unadjusted incidence rate was 2.4 cases/1,000 person-years at risk during the entire life course, and 11.0 cases/1,000 person-years at risk after blood draw. In comparison, 221 individuals developed a first MI in ULSAM. The unadjusted incidence rate was 3.0 cases/1,000 person-years at risk during the entire life course, and 10.8 cases/1,000 person-years at risk after blood draw.

### Associations of lipid-related SNPs with MI incidence

There were 10 SNPs that reached a nominal P-value of 0.01 or less for association with MI incidence in age- and sex-adjusted Cox models (**Table 2** **, left panel**) in STR. These SNPs were located in or near *CELSR2/SORT1, SREBF1, ABCA1, APOA1/APOC3/APOA5, SCARB1*and *LRP1*. The hazard ratios ranged from 0.79–0.81 and 1.20–1.40 per additional minor allele. None of the associations remained statistically significant after correcting for multiple testing using bootstrap re-sampling.

**Table 2:** Associations of genetic loci with MI in primary and replication analyses.

			STR (n = 2,602)			ULSAM (n = 1,142)		
SNPα	Nearest gene	Allelesε	HR	95% CI	P-value	HR	95% CI	P-value
rs4970834	*CELSR2* γ	A/G	1.30	1.11, 1.52	0.001	0.97	0.74, 1.27	0.818
rs9899634β	*SREBF1*	T/A	0.79	0.68, 0.93	0.003	0.96	0.78, 1.18	0.722
rs4149313	*ABCA1*	G/A	1.36	1.10, 1.67	0.004	1.40	1.07, 1.83	0.015
rs602633	*SORT1* γ	A/C	1.25	1.07, 1.47	0.005	0.94	0.75, 1.17	0.573
rs838900	*SCARB1*	G/A	1.40	1.10, 1.78	0.007	1.22	0.85, 1.74	0.281
rs3183702β	*SREBF1*	A/G	0.81	0.69, 0.94	0.007	0.99	0.81, 1.22	0.941
rs5090δ	*APOA4* γ	C/G	1.40	1.09, 1.80	0.009	NA	NA	NA
rs646776	*CELSR* γ	G/A	1.23	1.05, 1.44	0.010	0.97	0.78, 1.22	0.810
rs611917	*CELSR* γ	G/A	1.20	1.05, 1.39	0.010	0.91	0.74, 1.12	0.373
rs11172106β	*LRP1*	G/C	1.21	1.05, 1.39	0.010	1.10	0.91, 1.33	0.306

α Only SNPs associated with MI with a nominal P<0.01 in STR are presented. Data are hazard ratios with 95% confidence intervals and P-values from Cox proportional hazards regression models using age as time scale and study entry at age 18, adjusting for sex. The effect allele was defined as the minor allele in our study.

β Proxies rs2236513, rs4759275, and rs8066560 were used instead of lead SNPs rs3183702, rs11172106 and rs9899634, respectively in ULSAM (r^2^ = 1, 0.84 and 1) in all the results from the replication study.

γ CELSR2 and SORT1 reside in a cluster consisting of CELSR2/PSRC1/SORT1; APOA4 resides in a cluster of apolipoproteins APOA5/APOA4/APOC3/APOA1.

δ rs5090 was not successfully genotyped in ULSAM.

ε Alleles are presented as effect/other alleles.

Abbreviations: SNP, single nucleotide polymorphism; HR, hazard ratio; CI, confidence interval.

**Figure 1:**
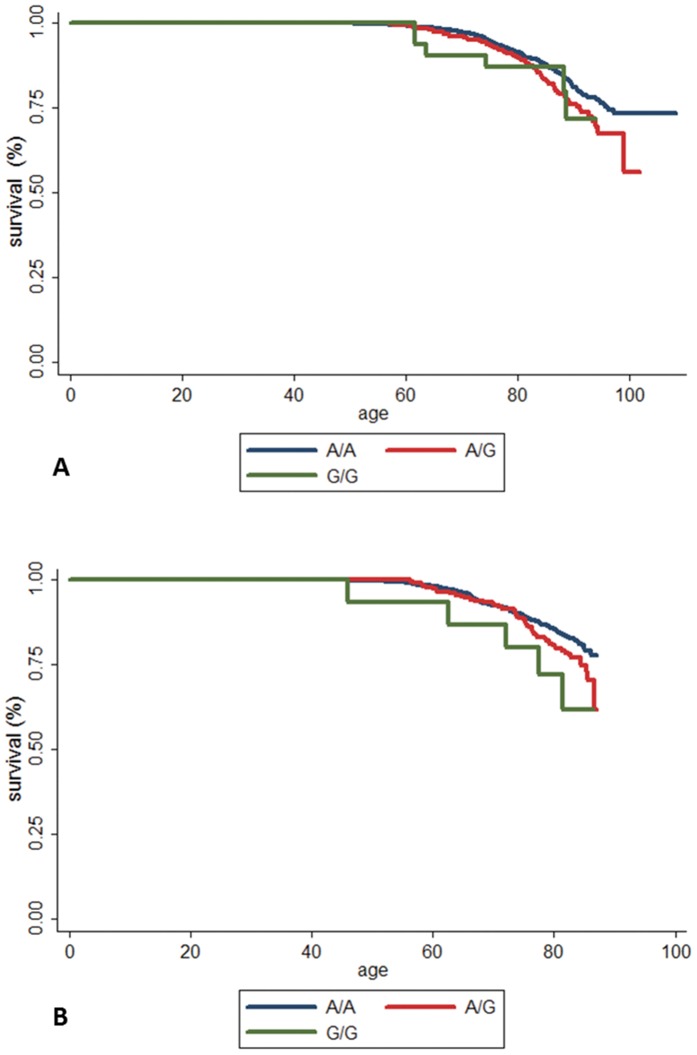
Kaplan-Meier survival function for rs4149313 and survival of MI in STR (A) and ULSAM (B). Survival function of MI for individuals having no (A/A; blue line); one (A/G; red line) or two (G/G; green line) risk alleles at *ABCA1* rs4149313 locus.

Of the 10 SNPs taken forward to replication in ULSAM, nine were successfully genotyped and passed our QC criteria. Of these, *ABCA1* rs4149313 demonstrated association with MI incidence in both study samples (**Table 2**). Each copy of the increasing effect allele of rs4149313 increased the risk of MI with 40% in ULSAM, and 36% in STR. Kaplan-Meier survival analysis for rs4149313 and MI showed that MI survival started to differ at age 70 in both STR (**Figure 1A**) and ULSAM (**Figure 1B**), indicating that the rs4149313 variant may predispose for MI later in life. The proportional hazard function for rs4149313 and MI association was fulfilled in both STR and ULSAM (P-values 0.821 and 0.651, respectively).

**Figure 2:**
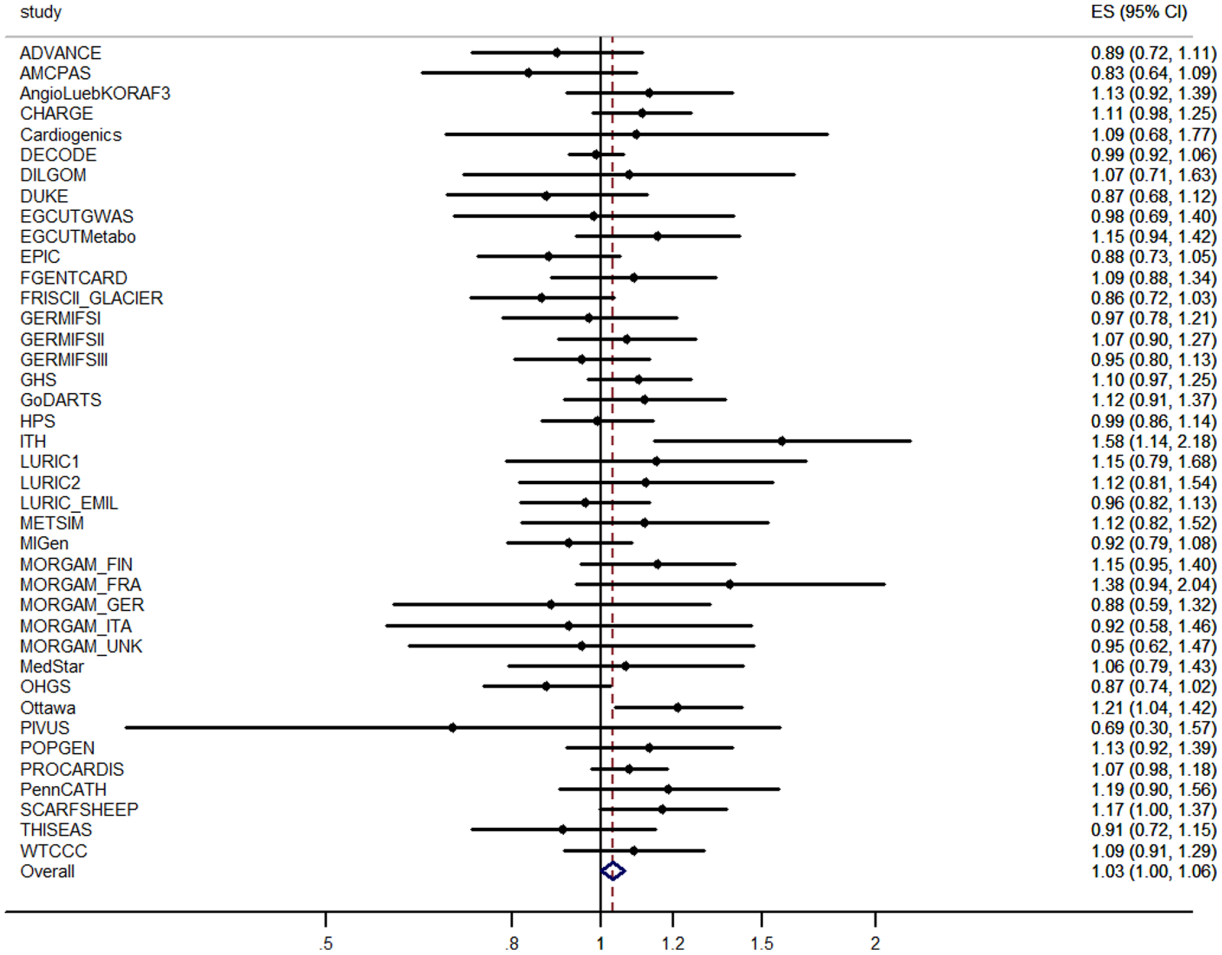
Forest plot for independent replication of association between rs4149313 and CAD in CARDIoGRAMplusC4D. Fixed-effect meta-analysis was applied for effect of rs4149313 on CAD in CARDIoGRAMplusC4D consortium. The diamond in the bottom represents the confidence interval of this independent replication.

After multiple testing corrections, the association between rs4149313 and MI incidence in ULSAM was not statistically significant (P-value, 0.135). To provide further evidence of an association with CAD, we performed *in silico* replication in a fixed-effects meta-analysis including 173,975 individuals of European descent from the CARDIoGRAMplusC4D consortium, where a SNP (rs4149311) in perfect LD with our lead SNP (rs4149313) showed the same effect direction with nominal significance (**Figure 2**; OR, 1.03; CI, 1.00–1.06; P-value 0.048).

### Additional analyses

We took into account the relatedness within each twin pair by fitting Cox models with shared frailty in STR. The estimates were broadly similar but had larger standard errors. (**Supplementary** **Table 2** **in File S1**).

Furthermore, in sensitivity analyses to exclude survival bias or left truncation of data as an explanation of our findings, we examined the associations in models with time at DNA collection as entry into the study. For these analyses, the median follow-up times were 9.0 years (249 events) in STR, and 11.3 years (122 events) in ULSAM. Due to fewer events and lower statistical power in these analyses, the confidence intervals were wider, and the association of *ABCA1* rs4149313 with incident MI was not statistically significant in STR, whereas it remained significant in ULSAM (HR, 1.11 and 1.56; 95% CI 0.84–1.47 and 1.10–2.23; P-value = 0.46 and 0.01 in STR and ULSAM, respectively) (**Supplementary Table 3 in File S1**).

The sex-specific analysis in STR showed that rs4149313 was significantly associated with MI in men (HR = 1.40, CI: 1.07, 1.83, p = 0.014); but not in women (HR = 1.28, CI: 0.92, 1.77, p = 0.137; **Supplementary Table 4 in File S1**). Associations between the 10 SNPs associated with MI and circulating lipid levels were also analyzed. None of the associations between SNPs and circulating lipid levels were consistently significant in both STR and ULSAM (**Supplementary Table 5 in File S1**).

*ABCA1* is a long gene, in which there were 47 SNPs genotyped (Supplementary Table 1 in File S1). Including rs4149313, there were 4 SNPs associated with MI at a nominally significant level (<0.05): rs4149313 (HR 1.36, P-value 0.004), rs2575879 (HR 1.19, P-value 0.014), rs2740487 (HR 1.16, P-value 0.034), rs10820743 (HR 0.85, P-value 0.045). As only rs4149313 among these four SNPs reached the threshold (nominal p<0.01) for further replication and additional analyses, we choose to bring only this variant forward. However, all of the four SNPs are potentially associated with MI, and further studies with larger sample sizes could be performed to investigate this possibility.

## Discussion

In the present study, we examined potential associations between 554 SNPs in 41 candidate gene regions proposed to be involved in lipid metabolism and MI incidence. We observed low nominal P-values (less than 0.01) for 10 genetic polymorphisms in our discovery sample. One of these associations, rs4149313 in the *ABCA1* region, was consistent in two cohorts and one meta-analysis of studies from CARDIoGRAMplusC4D consortium including 177,719 European individuals in total.

**Figure 3:**
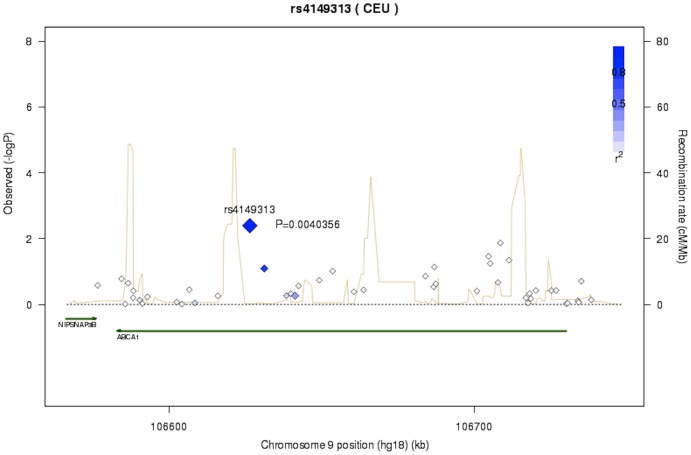
Region plot of the *ABCA1* locus (chromosome 9 co-ordinates 106157871–107157392) in the Swedish Twin Registry. The SNP rs4149313 with the strongest association is marked by a bright blue diamond. Each rhomb represents a SNP and the brightness represents the extent of linkage disequilibrium with the lead SNP. The recombination rates are marked with yellow lines. Relevant genes and genomic coordinates are shown below the plots. Plots were generated using SNAP (http://www.broadinstitute.org/mpg/snap/ldplot.php. Accessed 2013 Mar 1.) and based on HapMap CEU release 22, NCBI B36 assembly, dbSNP build 126.

*ABCA1*, which is located on chromosome 9q31.1, codes for a cellular phospholipid and cholesterol transporter that mediates the formation of nascent HDL particles. This gene has an important role in HDL metabolism and certain mutations could lead to Tangier disease [16]. rs4149313 is a non-synonymous SNP located in exon 18 of *ABCA1*, which encodes an amino acid substitution, Ile>Met (Ile883Met). Ile883Met is located at N-terminal of the first nucleotide-binding motif, suggesting its potential function on formatting stable structure of ATP-binding cassette. *In vitro* experiments showed that cholesterol efflux in transfected 293 cells with the I883M variant significantly differed from wild-type cells[20], which supports the biological functionality of this variant. Furthermore, this variant is conserved in different species, including zebra fish, western clawed frog, chicken, common turkey, mouse, rat, guinea pig, dog, bovine, pig, and lowland gorilla[21]. The conservation of this variant also indicates its importance, as well as offers opportunities for functional studies of rs4149313 in different model animals. As rs4149313 is situated in a tight LD block in *ABCA1* (**Figure 3**), further fine-mapping using a re-sequencing approach target on rare causal variants in the region may also prove useful to further understand the role of *ABCA1* in MI development.

The analyses in STR showed that the minor allele of rs4149313 increased risk of MI in Swedish populations while not being associated with circulating HDL levels. This finding is in line with a prior study by Frikke-Schmidt and colleagues[22] in a Danish population. The recent study from the Global Lipids Genetics Consortium [3] showed that the same allele that increased risk of MI/CAD in our study and in the prior Danish study also increase HDL and total cholesterol levels. This may seem conflicting, but recent Mendelian randomization studies[23], [24] have indicated that plasma HDL cholesterol levels may not be a causal factor for CAD. Also, the majority of variants previously reported to be associated with both HDL and CAD have pleiotropic effects[3]. For example, *CETP* variants are associated with decreasing plasma HDL and increasing LDL levels[25], [26]. Considering that *ABCA1* rs4149313 is also related with increasing total cholesterol level, it could affect CAD due to pleiotropic effects on other lipid fractions than HDL.

Several lipid-related loci have been identified in recent GWAS studies on MI/CAD, but cannot be replicated in our discovery study sample, for example, the *LDLR* and *PCSK9* loci[3], [27]. The reason for this is that the effect of genetic loci is small, and that our discovery sample size is modest, leading to low statistical power. This illustrates the need for great caution when interpreting our negative results, as larger samples are needed to refuse associations.

We report evidence of a weak association between the *ABCA1* locus and MI across three independent study samples including one meta-analysis of GWAS. The minor allele was associated with ∼40% increased risk of incident MI during follow-up in two longitudinal cohorts while 3% increased odds in the CARDIoGRAMplusC4D consortium. This difference in effect sizes could reflect inflated effect estimates in the discovery sample (Winner's curse) or some degree of between-study heterogeneity for example due to differences in study design, age groups or ethnicity. In a sensitivity analysis, we rerun association analyses of rs4149313 and coronary heart disease (including also unstable angina, percutaneous transluminal coronary angioplasty and coronary artery bypass graft surgery in the endpoint definition) in our discovery sample, STR. These analyses were very similar to our main analyses in STR (HR, 1.41; 95% CI, 1.15–1.72). Since the association between *ABCA1* rs4149313 and CAD showed such a weak effect (OR, 1.03) and just barely nominal significance (p = 0.048), we cannot not entirely rule out the possibility for this finding to be a false positive. However, as rs4149313 is a missense variant predicted to be potentially functional and given our two-stage replication design with consistent findings (albeit decreasing effect sizes), we believe our finding to be of interest and likely a true positive.

In Sweden, all medical care is publically funded and available to all citizens, thus all in-patient care, medication and death information can be easily followed through national registries. In the present study, we decided to investigate the first incident MI through participants' entire life course via combining information from the Swedish national registries with information from questionnaires and blood sampling results in our studies. Therefore, we could include cases even before those individuals participated in STR or ULSAM which increased power of the present study, and avoided recall bias from self-reporting of disease. However, as the Swedish Patient Register was not completely introduced nationally until 1987, those who had their first incident MI before 1987 would be missed if MI was never recurrent again. Since MI is a disease developed across a long period, occurring during old age and repeatedly, we consider the use of age 18 as the entry age as a proper way to deal with our data maximizing the statistical power. In additional sensitivity analyses, the association of rs4149313 with incident MI was weaker when using blood drawing time as entering of follow up. This could be due to a potential survival bias caused by those whose first MI incidence occurred before 1987, or due to decreased power by losing 37% of the cases compared to our main analysis. Individuals experiencing fatal MI cases before baseline could for natural reasons not enter our study. The potential survival bias introduced by such early onset fatal cases could potentially decrease the effect size of our findings, and drive them towards the null.

The strengths of present study include the use of two cohorts and one meta-analysis of GWAS, which allowed independent replication; a large number of genetic loci studied in relevant gene regions and the stringent criteria for claiming association. There are also some limitations of our study. First, statistical power in our discovery sample and longitudinal replication sample was fairly low, which resulted in a non-significant association of *ABCA1* rs4149313 with MI in STR and ULSAM after correction for multiple testing. Nevertheless, this association is replicated in two other independent study samples. The modest statistical power also means that several of our findings in the discovery sample that could not replicated could represent true associations with MI, and may merit further exploration in future studies. Second, candidate genes in this study were selected based on publications between 2003 to 2008 (largely before the GWAS era), and hence, many lipid-related loci that have been identified in later GWAS are not included in this study. Effects of such lipid loci on CHD would have to be explored in future studies. Third, since our study populations consisted of middle-aged to elderly individuals of European decent, the generalizability of our findings to younger individuals and other ethnicities is unknown.

## Conclusion

In conclusion, after investigation of genetic variation in genes from lipid-related pathways in two population-based cohorts and a meta-analysis of 173,975 individuals, we report *ABCA1* rs4149313 to be weakly associated with incidence of MI and risk of symptomatic CAD.

## Supporting Information

File S1 Supplementary Table 1, Candidate genes and SNPs included in the present study. Supplementary Table 2, Sensitivity analyses taking familial clustering into account. Supplementary Table 3, Sensitivity analyses using time at DNA collection as study entry after exclusion of individuals with any cardiovascular diseases before DNA collection. Supplementary Table 4, Sex-specific results in STR. Supplementary Table 5, Associations with lipids in STR and ULSAM.
(PDF)

## References

[1] Wilson PW, D'Agostino RB, Levy D, Belanger AM, Silbershatz H, et al (1998) Prediction of coronary heart disease using risk factor categories. Circulation 97: 1837–1847. 9603539 10.1161/01.cir.97.18.1837

[2] Daniels TF, Killinger KM, Michal JJ, Wright RW Jr, Jiang Z (2009) Lipoproteins, cholesterol homeostasis and cardiac health. Int J Biol Sci 5: 474–488. 19584955 10.7150/ijbs.5.474 PMC2706428

[3] Teslovich TM, Musunuru K, Smith AV, Edmondson AC, Stylianou IM, et al (2010) Biological, clinical and population relevance of 95 loci for blood lipids. Nature 466: 707–713. 20686565 10.1038/nature09270 PMC3039276

[4] Austin MA, Hutter CM, Zimmern RL, Humphries SE (2004) Familial hypercholesterolemia and coronary heart disease: a HuGE association review. Am J Epidemiol 160: 421–429. 15321838 10.1093/aje/kwh237

[5] Lichtenstein P, De Faire U, Floderus B, Svartengren M, Svedberg P, et al (2002) The Swedish Twin Registry: a unique resource for clinical, epidemiological and genetic studies. J Intern Med 252: 184–205. 12270000 10.1046/j.1365-2796.2002.01032.x

[6] Gold CH, Malmberg B, McClearn GE, Pedersen NL, Berg S (2002) Gender and health: a study of older unlike-sex twins. J Gerontol B Psychol Sci Soc Sci 57: S168–176. 11983743 10.1093/geronb/57.3.s168

[7] McClearn GE, Johansson B, Berg S, Pedersen NL, Ahern F, et al (1997) Substantial genetic influence on cognitive abilities in twins 80 or more years old. Science 276: 1560–1563. 9171059 10.1126/science.276.5318.1560

[8] Pedersen NL, Friberg L, Floderus-Myrhed B, McClearn GE, Plomin R (1984) Swedish early separated twins: identification and characterization. Acta Genet Med Gemellol (Roma) 33: 243–250. 6540957 10.1017/s0001566000007285

[9] Gatz M, Fratiglioni L, Johansson B, Berg S, Mortimer JA, et al (2005) Complete ascertainment of dementia in the Swedish Twin Registry: the HARMONY study. Neurobiol Aging 26: 439–447. 15653172 10.1016/j.neurobiolaging.2004.04.004

[10] Reynolds CA, Hong MG, Eriksson UK, Blennow K, Bennet AM, et al (2009) A survey of ABCA1 sequence variation confirms association with dementia. Hum Mutat 30: 1348–1354. 19606474 10.1002/humu.21076 PMC2758418

[11] HapMap Homepage. Available: http://hapmap.ncbi.nlm.nih.gov/index.html.en. Accessed 2013 Feb 28.

[12] Ingelsson E, Sundstrom J, Arnlov J, Zethelius B, Lind L (2005) Insulin resistance and risk of congestive heart failure. JAMA 294: 334–341. 16030278 10.1001/jama.294.3.334

[13] Lindblad U, Rastam L, Ranstam J, Peterson M (1993) Validity of register data on acute myocardial infarction and acute stroke: the Skaraborg Hypertension Project. Scand J Soc Med 21: 3–9. 8469941 10.1177/140349489302100102

[14] Hammar N, Alfredsson L, Rosen M, Spetz CL, Kahan T, et al (2001) A national record linkage to study acute myocardial infarction incidence and case fatality in Sweden. Int J Epidemiol 30 Suppl 1S30–34. 11759848 10.1093/ije/30.suppl_1.s30

[15] Barrett JC, Fry B, Maller J, Daly MJ (2005) Haploview: analysis and visualization of LD and haplotype maps. Bioinformatics 21: 263–265. 15297300 10.1093/bioinformatics/bth457

[16] Brooks-Wilson A, Marcil M, Clee SM, Zhang LH, Roomp K, et al (1999) Mutations in ABC1 in Tangier disease and familial high-density lipoprotein deficiency. Nat Genet 22: 336–345. 10431236 10.1038/11905

[17] Andrikovics H, Pongracz E, Kalina E, Szilvasi A, Aslanidis C, et al (2006) Decreased frequencies of ABCA1 polymorphisms R219K and V771M in Hungarian patients with cerebrovascular and cardiovascular diseases. Cerebrovasc Dis 21: 254–259. 16446539 10.1159/000091223

[18] Williams RL (2000) A note on robust variance estimation for cluster-correlated data. Biometrics 56: 645–646. 10877330 10.1111/j.0006-341x.2000.00645.x

[19] Deloukas P, Kanoni S, Willenborg C, Farrall M, Assimes TL, et al.. (2012) Large-scale association analysis identifies new risk loci for coronary artery disease. Nat Genet. 10.1038/ng.2480 PMC3679547 23202125

[20] Brunham LR, Singaraja RR, Pape TD, Kejariwal A, Thomas PD, et al (2005) Accurate prediction of the functional significance of single nucleotide polymorphisms and mutations in the ABCA1 gene. PLoS Genet 1: e83. 16429166 10.1371/journal.pgen.0010083 PMC1342637

[21] PolyPhen-2. Available: http://genetics.bwh.harvard.edu/pph2/index.shtml. Accessed 2013 Feb 28.

[22] Frikke-Schmidt R, Nordestgaard BG, Jensen GB, Steffensen R, Tybjaerg-Hansen A (2008) Genetic variation in ABCA1 predicts ischemic heart disease in the general population. Arterioscler Thromb Vasc Biol 28: 180–186. 17951323 10.1161/ATVBAHA.107.153858

[23] Voight BF, Peloso GM, Orho-Melander M, Frikke-Schmidt R, Barbalic M, et al (2012) Plasma HDL cholesterol and risk of myocardial infarction: a mendelian randomisation study. Lancet 380: 572–580. 22607825 10.1016/S0140-6736(12)60312-2 PMC3419820

[24] Haase CL, Tybjaerg-Hansen A, Qayyum AA, Schou J, Nordestgaard BG, et al (2012) LCAT, HDL cholesterol and ischemic cardiovascular disease: a Mendelian randomization study of HDL cholesterol in 54,500 individuals. J Clin Endocrinol Metab 97: E248–256. 22090275 10.1210/jc.2011-1846

[25] Thompson A, Di Angelantonio E, Sarwar N, Erqou S, Saleheen D, et al (2008) Association of cholesteryl ester transfer protein genotypes with CETP mass and activity, lipid levels, and coronary risk. JAMA 299: 2777–2788. 18560005 10.1001/jama.299.23.2777

[26] Gotto AM Jr, Moon JE (2012) Safety of inhibition of cholesteryl ester transfer protein with anacetrapib: the DEFINE study. Expert Rev Cardiovasc Ther 10: 955–963. 23030283 10.1586/erc.12.82

[27] Aulchenko YS, Ripatti S, Lindqvist I, Boomsma D, Heid IM, et al (2009) Loci influencing lipid levels and coronary heart disease risk in 16 European population cohorts. Nat Genet 41: 47–55. 19060911 10.1038/ng.269 PMC2687074

