# Supplementary material for: Genetic Variants from Lipid-Related Pathways and Risk for Incident Myocardial Infarction
Source: PLoS One. 2013 Mar 29;8(3):e60454. doi: 10.1371/journal.pone.0060454 (PMC3612051; doi:10.1371/journal.pone.0060454)
Supplement: File S1 — Supplementary Table 1, Candidate genes and SNPs included in the present study. Supplementary Table 2, Sensitivity analyses taking familial clustering into account. Supplementary Table 3, Sensitivity analyses using time at DNA collection as study entry after exclusion of individuals with any cardiovascular diseases before DNA collection. Supplementary Table 4, Sex-specific results in STR. Supplementary Table 5, Associations with lipids in STR and ULSAM. (PDF) [file pone.0060454.s001.pdf]

Supplementary Table 1. Candidate genes and SNPs included in the present study

Gene	SNP
PLA2G2A	rs2795480, rs13375942, rs6672057, rs4655020, rs1891320, rs12732308, rs10916683, rs11677, rs955587, rs2307246, rs2236771, rs11573142, rs10916685, rs12029284, rs7530853, rs1395470
PCSK9	rs11588151, rs2495498, rs2479417, rs17192725, rs2495488, rs17111495, rs2149041, rs2479409, rs4927193, rs529787, rs11206514, rs572512, rs585131, rs483462, rs615563, rs662145, rs13312, rs17410294, rs10493176
CELSR2/SORT1	rs4970834, rs611917, rs646776, rs602633, rs413582, rs464218, rs17585355, rs2303873, rs11142, rs11581665, rs12037569
GALNT2	rs6541294, rs4846908, rs1998064, rs10127775, rs4846918, rs609526, rs1124110, rs678050, rs611229, rs612577, rs4846928, rs4846930, rs4846935, rs11122469, rs3811488, rs3811486, rs2273966, rs656029, rs632557, rs16851269, rs3811485, rs7518564, rs2279432, rs4846849, rs1471915, rs9432145
APOB	rs1042034, rs1042031, rs1800479, rs1801701, rs693, rs1041968, rs11126598, rs11676704, rs679899, rs570877, rs1469513, rs1367117, rs588245, rs585967, rs17398765, rs11892073, rs934198, rs562338,

	rs754523
GTF3C2	rs1049817, rs11689803
GCKR	rs11675428, rs1260345, rs813592, rs8179219, rs1260326, rs3817588, rs780094, rs1919127
XAB1	rs4666009, rs2141371, rs10201652
IGF2BP2	rs11705729, rs4402960
HMGCR	rs10474433, rs3895886, rs3843480, rs3761739, rs17244953, rs2241402, rs11742194, rs12654264, rs3846662, rs4629571
AGER	rs2269423, rs9469089, rs3134943, rs1035798, rs1800684, rs1800625, rs204995, rs204993, rs3132940
PLA2G7	rs974670, rs12528857, rs1051931, rs2216465, rs4498351, rs1421372, rs17288905, rs16874962, rs1805017, rs3799861, rs9472830, rs12528807, rs1421368, rs1421378, rs1421379, rs1862008, rs12211537, rs9296510
ACAT2	rs1475123, rs927450, rs25683, rs3465, rs15982, rs4832, rs2295898
TBL2	rs17145738, rs35607697, rs2286276
MLXIPL	rs11772762, rs6967107, rs3812316, rs7798357, rs17145813, rs13230514, rs799166, rs7777102
CHRM2	rs2350780, rs2061174, rs7799047, rs2350786, rs6948054, rs324650, rs8191992, rs1378650, rs1424548

LPL	rs17410577, rs1534649, rs3779788, rs248, rs249, rs253, rs255, rs256, rs263, rs270, rs281, rs283, rs285, rs301, rs316, rs320, rs328, rs10099160, rs11570891, rs1059507, rs2197089, rs10105606, rs2410617, rs10503669
CDKN2A/B	rs2383208, rs10811661
ABCA1	rs2515606, rs363717, rs4149338, rs2482433, rs2740485, rs2066881, rs4149336, rs2297406, rs2020927, rs2230808, rs2297404, rs2777801, rs2066716, rs2297409, rs4149313, rs2066717, rs2065412, rs2515601, rs2472386, rs2274873, rs4149291, rs1175293, rs2230806, rs2230805, rs4149271, rs11789603, rs4149268, rs3890182, rs10120087, rs2740487, rs2777793, rs3905001, rs2575879, rs10820743, rs4149264, rs4149262, rs12350560, rs2515618, rs2437811, rs10991414, rs2472377, rs1800977, rs2246293, rs2043664, rs2487042, rs2472496, rs3887137
ABCA2	rs6560653
IDE/HHEX	rs2149632, rs7084090, rs1111875, rs5015480, rs7087591, rs7923837
SAA4/SAA2/SAA1	rs7940510, rs2445227, rs2445176, rs11024578, rs1520887, rs1520886, rs12282742, rs7113375, rs7130337, rs3825023, rs7108343, rs10832911, rs1993373, rs11603089, rs4638289, rs1829575, rs11024600
BDNF	rs7124442, rs6265, rs11030104, rs2049045, rs7103411, rs7104207, rs1013402, rs7127507,

	rs2030324, rs12273363, rs908867
NR1H3	rs4752973, rs7118396, rs10838681, rs11039149, rs3758674, rs12221497, rs2279238, rs7114704, rs10501320, rs10501321, rs2291119
ACAT1	rs15677, rs12577790, rs7114794, rs7931681, rs10890817, rs11607588, rs35188041, rs3741049, rs10890818, rs11212524, rs10890819, rs2280332, rs4754298
APOA5/APOA4/APOC3/APOA1	rs28927680, rs12286037, rs619054, rs2072560, rs3135506, rs662799, rs1729409, rs1263167, rs1263173, rs5110, rs5104, rs5092, rs5090, rs2854117, rs4520, rs689243, rs888245, rs625145
SORL1	rs668387, rs689021, rs641120, rs2070045, rs3824968, rs2282649
LRP1	rs324013, rs11172106, rs12298170, rs715948, rs4759277, rs1799986, rs7398375, rs10876966, rs1800194, rs12814239, rs1800139, rs1800154, rs1800156, rs1800168, rs1140648, rs1800159, rs7956957
SCARB1	rs838879, rs838883, rs701106, rs838887, rs838895, rs838896, rs5888, rs989892, rs865716, rs838900, rs4765615, rs745529, rs1902569, rs11608336, rs12581963, rs10773107, rs10744182, rs4765622, rs10773109, rs12370382, rs3924313, rs10773111, rs11615630, rs4379922, rs10846760, rs6488950, rs7295752
CYP46A1	rs2895812, rs4145039, rs943884, rs10137574, rs943880, rs2146238, rs2273839, rs12434024,

	rs3752958, rs7157032, rs1957514, rs8018381, rs2281682
LIPC	rs4775041, rs4774297, rs1800588, rs6494005, rs11632618, rs8034802, rs166362, rs261337, rs634746, rs488490, rs3825776, rs10518976, rs934297, rs12909642, rs7182229, rs877480, rs17190587, rs1869137, rs1973028, rs1869144, rs4613005, rs4775067, rs12904012, rs11853726, rs2233741, rs2242062, rs690, rs16940472, rs6083, rs2242065, rs7165654, rs7166788, rs871804, rs7178362, rs17190678, rs3751542, rs17269397, rs6074
SELS	rs4965811, rs12910524, rs9806366, rs2101171, rs9874, rs4965373, rs4965814, rs10152335, rs4965820
CETP	rs247611, rs9989419, rs6499863, rs12708967, rs3764261, rs12447924, rs17231506, rs1800775, rs1864163, rs9929488, rs7203984, rs7205804, rs1532625, rs12708974, rs9930761, rs5883, rs289717, rs12720898, rs5880, rs1800774, rs5882, rs1801706, rs289742, rs289745, rs17369163, rs12934552
LCAT	rs17240392, rs1109166, rs2292318
SREBF1	rs11653007, rs9899634, rs3183702
LIPG	rs12962061, rs2000812, rs2000813, rs2097055, rs8093249, rs2276269, rs9951026, rs11664186, rs12604221, rs12606544, rs4939585, rs16950701, rs2156552
LDLR	rs6511720, rs8110695, rs2228671, rs2569559, rs1003723, rs5930, rs1569372, rs4508523, rs2738446,

	rs2738447, rs1799898, rs5925, rs5927, rs2569540, rs12459476, rs2738460, rs2738464
APOE	rs429358, rs7412
NR1H2	rs11666394, rs17373080, rs4802703
PLTP	rs7270170, rs1736493, rs553359, rs11086985, rs435306, rs441346, rs394643, rs2294212, rs17447545, rs6065906, rs6032592
SREBF2	rs5758487, rs1009544, rs2284082, rs2413660, rs2839713, rs133280, rs17002706, rs11702960, rs4822063, rs1052717, rs17002737, rs2267443, rs4822066, rs713881, rs2269661

Supplementary Table 2. Sensitivity analyses taking familial clustering into account

SNP	HR	95% CI	P-value
rs4970834	1.30	1.10 , 1.53	0.002
rs9899634	0.79	0.68 , 0.93	0.003
rs4149313	1.36	1.10 , 1.67	0.004
rs602633	1.25	1.06 , 1.47	0.007
rs838900	1.40	1.09 , 1.79	0.009
rs3183702	0.81	0.69 , 0.94	0.007
rs5090	1.40	1.08 , 1.81	0.011
rs646776	1.23	1.04 , 1.44	0.013
rs611917	1.20	1.04 , 1.39	0.011
rs11172106	1.21	1.04 , 1.40	0.012

Associations between SNPs and MI in STR, adjusted for twin relatedness using Cox proportional hazards regression with age as time scale and study entry at age 18, adjusting sex and familiar clustering. The effect allele was defined as the minor allele in our study. Results are reported as hazard ratios with 95% confidence intervals and P-values. Only SNPs at loci predominantly associated with MI in STR (Table 2) were reported.

Abbreviations: SNP, single nucleotide polymorphism; HR, hazard ratio; CI, confidence interval.

Supplementary Table 3. Sensitivity analyses using time at DNA collection as study entry after exclusion of individuals with any cardiovascular diseases before DNA collection

	**STR (n=2,436)**			**ULSAM (n=1,082)**		
SNP	HR	95% CI	P-value	HR	95% CI	P-value
rs4970834	1.08	0.88, 1.33	0.449	0.88	0.61 , 1.27	0.500
rs9899634	0.88	0.64, 1.09	0.191	1.02	0.78 , 1.34	0.892
rs4149313	1.11	0.84, 1.47	0.457	1.56	1.10 , 2.24	0.014
rs602633	1.10	0.90, 1.35	0.340	0.96	0.71 , 1.29	0.782
rs838900	1.56	1.17, 2.08	0.002	1.11	0.68 , 1.82	0.680
rs3183702	0.90	0.74, 1.08	0.269	1.04	0.80 , 1.36	0.767
rs5090	1.65	1.22, 2.22	0.001	NA	NA	NA
rs646776	1.09	0.89, 1.34	0.387	1.00	0.74 , 1.34	0.987
rs611917	1.12	0.94, 1.34	0.203	0.87	0.66 , 1.15	0.325
rs11172106	1.05	0.88, 1.27	0.576	1.13	0.88 , 1.45	0.343

Associations between SNPs and MI in STR using Cox proportional hazards regression with age as time scale, study entry at blood withdrawal as entry time, adjusting for sex. The effect allele was defined as the minor allele in our study. Results are reported as hazard ratios with 95% confidence intervals and P-values. Only SNPs at loci associated with MI in STR (table 2) are reported.

Abbreviations: SNP, single nucleotide polymorphism; HR, hazard ratio; CI, confidence interval.

Supplementary Table 4. Sex-specific results in STR

	Male(n=1,124)			Female(n=1,478)		
SNP	HR	95% CI	P-value	HR	95% CI	P-value
rs4970834	1.22	0.99, 1.50	0.068	1.41	1.12, 1.79	0.004
rs9899634	0.71	0.58, 0.87	0.001	0.92	0.73, 1.17	0.509
rs4149313	1.40	1.07, 1.84	0.014	1.28	0.92, 1.77	0.137
rs602633	1.20	0.97, 1.48	0.090	1.34	1.05, 1.69	0.017
rs838900	1.40	1.03, 1.90	0.031	1.39	0.94, 2.06	0.097
rs3183702	0.72	0.58, 0.88	0.002	0.95	0.75, 1.20	0.638
rs5090	1.42	1.03, 1.95	0.033	1.38	0.91, 2.09	0.126
rs646776	1.17	0.95, 1.43	0.141	1.32	1.04, 1.67	0.021
rs611917	1.15	0.95, 1.38	0.149	1.29	1.04, 1.60	0.020
rs11172106	1.13	0.94, 1.37	0.194	1.32	1.05, 1.65	0.016

Associations between SNPs with MI in STR stratified by sex using Cox proportional hazards regression with age as time scale and study entry at age 18. The effect allele was defined as the minor allele in our study. Results are reported as hazard ratio with 95% confidence intervals and P-values. Only SNPs at loci predominantly associated with MI in STR (table 2) are reported here.

Abbreviations: SNP, single nucleotide polymorphism; HR, hazard ratio; CI, confidence interval.

Supplementary Table 5. Associations with lipids in STR and ULSAM

			STR				ULSAM			
SNP	Alleles (effect/other)	TRAITS	N	Beta	95% CI	P-value	N	Beta	95% CI	P-value
rs4970834	A/G	TC	2447	-0.170	-0.256 , -0.084	$1.122e^{-04}$	883	-0.062	-0.186 , 0.062	0.326
		HDL	2046	-0.021	-0.050 , 0.008	0.161	881	0.012	-0.031 , 0.056	0.575
		LDL	1609	-0.154	-0.244 , -0.065	0.001	878	-0.057	-0.168 , 0.054	0.313
		TG^{α}	2029	0.004	-0.031 , 0.039	0.826	883	-0.011	-0.070 , 0.048	0.707
		APOA1	1054	-0.002	-0.030 , 0.025	0.860	385	0.05	0.003 , 0.097	0.038
		APOB	1054	-0.048	-0.082 , -0.014	0.005	385	-0.019	-0.063 , 0.024	0.390
rs9899634	T/A	TC	2449	0.024	-0.054 , 0.102	0.543	911	0.006	-0.089 , 0.102	0.901
		HDL	2056	-0.012	-0.038 , 0.015	0.390	909	0.023	-0.011 , 0.058	0.185
		LDL	1617	-0.010	-0.090 , 0.071	0.810	906	-0.003	-0.089 , 0.082	0.938
		TG^{α}	2030	0.000	-0.032 , 0.032	0.985	911	-0.022	-0.068 , 0.023	0.337
		APOA1	1055	-0.011	-0.036 , 0.014	0.393	379	0.017	-0.018 , 0.052	0.342
		APOB	1055	-0.006	-0.037 , 0.026	0.716	379	-0.015	-0.047 , 0.018	0.377
rs4149313	G/A	TC	2500	0.010	-0.108 , 0.127	0.872	963	0.045	-0.093 , 0.182	0.524
		HDL	2095	0.032	-0.007 , 0.071	0.112	961	-0.025	-0.074 , 0.024	0.321
		LDL	1652	0.010	-0.109 , 0.128	0.875	958	0.053	-0.069 , 0.175	0.396
		TG^{α}	2077	-0.007	-0.054 , 0.041	0.788	963	0.038	-0.027 , 0.104	0.248
		APOA1	1094	0.019	-0.018 , 0.055	0.313	424	-0.040	-0.094 , 0.015	0.155
		APOB	1094	-0.011	-0.057 , 0.034	0.627	424	0.026	-0.025 , 0.076	0.320
rs602633	A/C	TC	2394	-0.174	-0.259 , -0.090	$5.470e^{-05}$	962	-0.028	-0.128 , 0.073	0.588
		HDL	2021	-0.012	-0.041 , 0.016	0.403	960	0.024	-0.012 , 0.060	0.190

		LDL	1593	-0.147	-0.233 , -0.062	0.001	957	-0.048	-0.138 , 0.041	0.291
		TG$^{\alpha}$	1986	0.000	-0.035 , 0.035	0.999	962	0.007	-0.041 , 0.055	0.770
		APOA1	1013	-0.002	-0.029 , 0.025	0.884	424	0.060	0.022 , 0.098	0.002
		APOB	1013	-0.051	-0.085 , -0.017	0.003	424	-0.002	-0.037 , 0.034	0.918
rs838900	G/A	TC	2498	0.196	0.055 , 0.337	0.006	956	0.097	-0.075 , 0.269	0.267
		HDL	2094	0.007	-0.040 , 0.055	0.759	954	0.005	-0.057 , 0.066	0.881
		LDL	1651	0.098	-0.048 , 0.244	0.190	951	0.034	-0.119 , 0.188	0.663
		TG$^{\alpha}$	2075	0.009	-0.048 , 0.067	0.753	956	0.082	0.001 , 0.164	0.049
		APOA1	1093	0.017	-0.026 , 0.061	0.440	421	-0.015	-0.073 , 0.043	0.608
		APOB	1093	0.039	-0.016 , 0.093	0.163	421	0.058	0.005 , 0.111	0.034
rs3183702	A/G	TC	2425	0.036	-0.042 , 0.114	0.367	878	-0.005	-0.101 , 0.091	0.913
		HDL	2027	-0.015	-0.041 , 0.012	0.270	877	0.023	-0.011 , 0.058	0.191
		LDL	1593	0.002	-0.079 , 0.083	0.956	874	-0.008	-0.093 , 0.078	0.861
		TG$^{\alpha}$	2010	0.001	-0.032 , 0.033	0.968	878	-0.031	-0.077 , 0.015	0.191
		APOA1	1037	-0.017	-0.042 , 0.009	0.192	404	0.002	-0.032 , 0.036	0.903
		APOB	1037	0.000	-0.031 , 0.032	0.982	404	-0.026	-0.057 , 0.005	0.104
rs5090	C/G	TC	2500	-0.005	-0.157 , 0.146	0.944	NA	NA	NA	NA
		HDL	2095	-0.021	-0.072 , 0.029	0.413	NA	NA	NA	NA
		LDL	1652	0.060	-0.094 , 0.213	0.445	NA	NA	NA	NA
		TG$^{\alpha}$	2077	-0.003	-0.065 , 0.059	0.926	NA	NA	NA	NA
		APOA1	1094	-0.024	-0.074 , 0.027	0.360	NA	NA	NA	NA
		APOB	1094	0.007	-0.056 , 0.070	0.828	NA	NA	NA	NA
rs646776	G/A	TC	2493	-0.185	-0.268 , -0.101	$1.510e^{-05}$	958	-0.024	-0.126 , 0.078	0.642
		HDL	2089	-0.014	-0.042 , 0.014	0.319	956	0.022	-0.014 , 0.058	0.240
		LDL	1647	-0.170	-0.256 , -0.084	$1.164e^{-04}$	953	-0.052	-0.143 , 0.039	0.267

		TG^{α}	2071	0.007	-0.028 , 0.041	0.701	958	0.019	-0.030 , 0.067	0.452
		APOA1	1091	0.000	-0.027 , 0.027	0.989	421	0.058	0.020 , 0.097	0.003
		APOB	1091	-0.045	-0.079 , -0.011	0.009	421	-0.002	-0.038 , 0.034	0.928
rs611917	G/A	TC	2488	-0.110	-0.184 , -0.036	0.004	962	-0.058	-0.151 , 0.035	0.221
		HDL	2084	-0.004	-0.029 , 0.021	0.759	960	0.022	-0.011 , 0.055	0.198
		LDL	1642	-0.070	-0.145 , 0.006	0.071	957	-0.081	-0.164 , 0.002	0.055
		TG^{α}	2066	-0.010	-0.040 , 0.021	0.538	962	$4.247e^{-04}$	-0.044 , 0.045	0.985
		APOA1	1090	-0.001	-0.025 , 0.023	0.909	423	0.045	0.011 , 0.079	0.009
		APOB	1090	-0.032	-0.062 , -0.002	0.036	423	-0.009	-0.041 , 0.022	0.571
rs11172106	G/C	TC	2497	0.020	-0.053 , 0.094	0.588	971	-0.064	-0.151 , 0.023	0.153
		HDL	2092	-0.006	-0.030 , 0.018	0.629	969	0.012	-0.019 , 0.044	0.446
		LDL	1649	-0.026	-0.100 , 0.049	0.497	966	-0.061	-0.139 , 0.017	0.127
		TG^{α}	2074	-0.011	-0.041 , 0.019	0.475	971	-0.035	-0.077 , 0.006	0.096
		APOA1	1094	-0.005	-0.029 , 0.019	0.676	428	-0.009	-0.040 , 0.023	0.59
		APOB	1094	0.002	-0.028 , 0.032	0.899	428	-0.02	-0.049 , 0.009	0.184

Associations between SNPs at loci associated with MI with serum lipids levels including TC, HDL, LDL, TG, APOA1 and APOB. Data are effect sizes (beta) with 95% confidence intervals and P-values from linear regressions assuming additive genetic models. Left panel shows results from STR analyzed with multilevel mixed-effects linear regression models adjusted by age, sex and fasting status. Right panel includes results from ULSAM analyzed with linear regression adjusted by age. The effect allele was defined as the minor allele in our study. Fasting here refers fasting overnight before taking blood tests for circulating lipids levels.

$^{\alpha}$We used natural log TG for TG trait.

Abbreviations: SNP, single nucleotide polymorphism; CI, confidence interval; TC, total cholesterol; HDL, high-density lipoprotein cholesterol; LDL, low-density lipoprotein cholesterol; TG, triglycerides; APOA1, apolipoprotein AI; APOB, apolipoprotein B.

Full author list and affiliation for CARDIoGRAMplusC4D consortium

Panos Deloukas[1], Stavroula Kanoni[1], Christina Willenborg[2-3], Martin Farrall[4-5], Themistocles L Assimes[6], John R Thompson[7,], Erik Ingelsson[8], Danish Saleheen[9-11], Jeanette Erdmann[2-3], Muredach P Reilly[12], Rory Collins[13], SekarKathiresan[14-15], Anders Hamsten[16-17], Unnur Thorsteinsdottir[18-19], Jaspal S Kooner[20], John Danesh[10], Colin Neil Alexander Palmer[21], Robert Roberts[22], Hugh Watkins[4-5], Heribert Schunkert[2-3], Nilesh J Samani[23-24], Stefan Blankenberg[25], Bernhard O Boehm[26], John C Chambers[27], Robert Clarke[13], George Dedoussis[28], Paul W Franks[29-30], Christian Hengstenberg[31], GKeesHovingh[32], Jong-Young Lee[33], TerhoLehtimäki[34-35], Winfried März[36-37], Andres Metspalu[38-39], Markku S Nieminen[40], Christopher O'Donnell[41], Markus Perola[42], SamuliRipatti[1, 22], Manjinder S Sandhu[1,43], Stefan Schreiber[44], Agneta Siegbahn[45], Cristen J Willer[46], Pierre A Zalloua[47], Benjamin A Goldstein[6], Kathleen Stirrups[1], Inke R König[48], Jean-Baptiste Cazier[4], Åsa Johansson[45],Tõnu Esko[38-39], Lasse Folkersen[16-17], Anuj Goel[4-5], Aki S Havulinna[42], Weang K Ho[10], Jemma C Hopewell[13], NiclasEriksson[45], Marcus E Kleber[37,49], Kati Kristiansson[42], Per Lundmark[50], Leo-Pekka Lyytikäinen[34-35], Suzanne Rafelt[23], Dmitry Shungin[29], Rona J Strawbridge[16-17], Gudmar Thorleifsson[18], Emmi Tikkanen[22], Natalie Van Zuydam[21], Benjamin F Voight[51], Lindsay L Waite[52], Weihua Zhang[27], Andreas Ziegler[48], Devin Absher[52], Ines Barroso[1,53], Christof Burgdorf[2-3], Simone Claudi-Boehm[54], DIAGRAM consortium, NourEddine El Mokhtari[55], Krista Fischer[38], Pierre Fontanillas[15], Bruna Gigante[56], Leif Groop[57], Stefan Gustafsson[8], Jörg Hager[58], Bok-Ghee Han[33], Sarah E Hunt[1], Thorsten Kessler[2,3], Joshua W Knowles[6], Claudia Langenberg[43], Cordelia Langford[1], Mark I McCarthy[4, 59-60], Martina Müller-Nurasyid[61-63], Kjell Nikus[64], John F Peden[4], Silke Rosinger[26], Diana Rubin[65], Moritz P Rumpf[2-3], Arne Schäfer[44], Alexandre FR Stewart[22], Peter J Wagner[66], Wellcome Trust Case Control Consortium; Hyun M Kang[67], N William Rayner[4], Elin Grundberg[1,68], Per Eriksson[16-17], Anders Franco-Cereceda[69], Anders Lundmark[50], Olle Melander[57], Seraya Maouche[70], MuTHER, Tsun-Po Yang[1], Alison H Goodall[23], Eric Schadt[71], TomiPastinen[72], Ann-Christine Syvänen[50], Alan S Go[73], Mark A Hlatky[6], Thomas Quertermous[6], Ellen C van der Schoot[74], HannekeBasart[32], Patrick Diemert[25], Philipp S Wild[73], CARDIOGENICS, Francois Cambien[70], Willem H Ouwehand[1,76-78], Eric Boerwinkle[78], Adrienne L Cupples[79], Abbas Dehghan[80], Vilmundur Gudnason[19,81], Marja-Liisa Lokki[82], Juha Sinisalo[40], Gudmundur Thorgeirsson[19,83], Hilma Holm[18], Kari Stefansson[18,83], Nicholas Wareham[43], Evelin Mihailov[39], Dominique Gauguier[4,84], Mika Kähönen[85], Veikko Salomaa[42], Lars Wallentin[45], Thomas Illig[86], Christa Meisinger[87], Klaus Stark[31], Martina E Zimmermann[31], Göran Hallmans[88], Alex SF Doney[21], Andrew D Morris[21], Sarah Parish[13], Yangsoo Jang[89], Hyo-Soo Kim[90], Ji-Young Lee[33], Jeong E Park[91], Sian-Tsung Tan[20], ReijoLaaksonen[92], Johanna Kuusisto[93], Michael Boehnke[67], Markku Laakso[93], Ron Do[15,94], Kari Kuulasmaa[42], Jarmo Virtamo[42], Philippe Amouye[l95], Dominique Arveiler[96], Jean Ferrières[97], David-AlexandreTrégouët[70], Norman Klopp[86], Annette Peters[87,98], Paolo Brambilla[99], Marco M Ferrario[100], AlunEvans[101], Frank Kee[102], George A Wells[22], Stanley L Hazen[103], SvatiShah[104], Stephen Epstein[105], Daniel J Rader[106], David Altshuler[15,107-108], David Cox[109], Ci Song[8], Lars Lind[50], Nancy L Pedersen[8], Asif Rasheed[9], Karin Leander[56], Ulf de Faire[56], Maria Dimitriou[28], Genovefa Kolovou[110], Ulm (EMIL) Wolfgang Koenig[111], Wolfgang Kratzer[111], Anthony JBalmforth[112], Peter S Braund[23-24], Mohan Sivananthan[113], Alistair S Hall[114]

1Wellcome Trust Sanger Institute, Hinxton UK; 2Universität zu Lübeck, Medizinische Klinik II, Ratzeburger Allee 160, 23538 Lübeck, Germany; 3 Deutsches Zentrum für Herz-Kreislauf-Forschung (DZHK), 23538 Lübeck, Germany; 4Wellcome Trust Centre for Human Genetics, University of Oxford, UK; 5Cardiovascular Medicine, University of Oxford, Oxford UK; 6 Department of Medicine, Stanford University School of Medicine; 7Department of Health Sciences, University of Leicester, Leicester UK; 8 Department of Medical Epidemiology and Biostatistics, Karolinska Institute, Stockholm, Sweden; 9Center for Non-Communicable Diseases, Karachi, Pakistan; 10Department of Public Health and Primary Care, University of Cambridge, UK; 11Department of Medicine, University of Pennsylvania, USA; 12 Cardiovascular Institute, Perelman School of Medicine at the University of Pennsylvania, Philadelphia, PA 19130, USA; 13Clinical Trial Service Unit and Epidemiological Studies Unit, University of Oxford, Oxford, UK; 14 Cardiology Division, Center for Human Genetic Research, and Cardiovascular Research Center, Massachusetts General Hospital and Harvard Medical School, Boston, USA; 15Broad Institute of Harvard/MIT, Cambridge, USA; 16 Atherosclerosis Res Unit, Department of Medicine, Karolinska Institutet; 17Center for Molecular Medicine, Karolinska University Hospital, Stockholm, Sweden; 18 deCODE genetics, Sturlugata 8, Reykjavik, Iceland; 19University of Iceland, Faculty of Medicine, Reykjavik, Iceland; 20 National Heart and Lung Institute (NHLI), Imperial College London, Hammersmith Hospital, London, UK; 21Medical Research Institute, University of Dundee, Ninewells Hospital and Medical School, Dundee, DD1 9SY UK; 22 Institute for Molecular Medicine FIMM, University of Helsinki and Public Health Genomics Unit, National Institute for Health and Welfare, Helsinki, Finland; 23 Department of Cardiovascular Sciences, University of Leicester, Glenfield Hospital, Leicester, LER 9QP, UK; 24National Institute for Health Research Leicester Cardiovascular Biomedical Research Unit, Glenfield Hospital, Leicester LE3 9QP, UK; 25University Heart Center Hamburg, Clinic for general and interventional Cardiology, Hamburg, Germany; 26Division of Endocrinology and Diabetes, Department of Internal Medicine, Ulm University Medical Centre, Ulm, Germany; 27Epidemiology and Biostatistics, Imperial College London, London, UK; 28Department of Dietetics-Nutrition, Harokopio University, 70 El. Venizelou Str, Athens, Greece; 29Department of Clinical Sciences, Genetic & Molecular Epidemiology Unit, Lund University Diabetes Center, Skåne University Hosptial, Malmö, Sweden & Department of Public Health & Clinical Medicine, Genetic Epidemiology & Clinical Research Group, Section for Medicine & Department of Odontology, Umeå University, Umeå, Sweden; 30Department of Nutrition, Harvard School of Public Health, Boston, MA USA; 31Klinik und Poliklinik für Innere Medizin II, Regensburg, Germany; 32Dept Vascular Medicine, Academic Medical Center, Amsterdam, The Netherlands; 33 Center for Genome Science, Korea National Institute of Health, KCDC, Chungcheongbuk-do 363-951, Korea; 34Department of Clinical Chemistry, Fimlab Laboratories, Tampere University Hospital, Tampere 33101, Finland; 35Department of Clinical Chemistry, University of Tampere School of Medicine, Tampere 33521, Finland ; 36Synlab Academy, Gottlieb-Daimler-Straße 25, 68165 Mannheim, Germany; 37Mannheim Institute of Public Health, Social and Preventive Medicine, Medical Faculty of Mannheim, University of Heidelberg, Ludolf-Krehl-Strasse 7-11, 68167 Mannheim,

Germany; 38Estonian Genome Center, University of Tartu, Tartu, Estonia; 39Insitute of Molecular and Cell Biology, University of Tartu, Tartu, Estonia; 40Division of Cardiology, Department of Medicine, Helsinki University Central Hospital (HUCH), Helsinki, Finland; 41Framingham Heart Study, National Heart, Lung and Blood Institute, Framingham, MA 01702, US; 42Department of Chronic Disease Prevention, National Institute for Health and Welfare, Helsinki FIN-00271, Finland; 43MRC Epidemiology Unit, Institute of Metabolic Science, Addenbrooke's Hospital, Box 285, Cambridge, CB2 0QQ, UK; 44Institut für Klinische Molekularbiologie, Christian-Albrechts Universität, Kiel, Germany; 45Uppsala Clinical Research Center, Uppsala University, Uppsala, Sweden; 46Department of Internal Medicine , Division of Cardiovascular Medicine, and Department of Human Genetics, University of Michigan, Ann Arbor, Michigan, USA; 47Lebanese American University, Chouran Beirut, Lebanon; 48Institut für Medizinische Biometrie und Statistik, Universität zu Lübeck, Lübeck, Germany; 49LURIC Study nonprofit LLC, Platz der alten Synagoge 1, 79098 Freiburg, Germany; 50 Department of Medical Sciences, Uppsala University, Uppsala, Sweden; 51Department of Pharmacology, University of Pennsylvania, Philadelphia, USA; 52HudsonAlpha Institute for Biotechnology, Huntsville, AL, USA; 53 University of Cambridge, Metabolic Research Laboratories, Institute of Metabolic Science, Addenbrooke's Hospital, Cambridge, UK; 54Practice of Gynecology, Ulm University Medical Centre, Ulm, Germany; 55Klinik für Innere Medizin, Kreiskrankenhaus Rendsburg, Rendsburg, Germany; 56Division of Cardiovascular Epidemiology, Institute of Environmental Medicine, Karolinska Institutet, Stockholm, Sweden; 57Department of Clinical Sciences, Diabetes and Endocrinology, Lund University, University Hospital Malmö, Malmö, Sweden.; 58CEA-Genomics Institute, National Genotyping Centre, Paris, France; 59Oxford Centre for Diabetes, Endocrinology and Metabolism, University of Oxford, OX3 7LJ UK; 60Oxford National Institute for Health Research Biomedical Research Centre, Churchill Hospital, Old Road Headington, Oxford, OX3 7LJ, UK; 61Department of Medicine I, University Hospital Grosshadern, Ludwig-Maximilians-Universität, Munich, Germany; 62 Institute of Medical Informatics, Biometry and Epidemiology, Chair of Epidemiology and Chair of Genetic Epidemiology, Ludwig-Maximilians-Universität, Munich, Germany; 63 Institute of Genetic Epidemiology, Helmholtz Zentrum München - German Research Center for Environmental Health, Neuherberg, Germany; 64 Heart Centre, Department of Cardiology, Tampere University Hospital, Tampere 33521, Finland; 65MedizinischeKlinik I, Universitätsklinikum Schleswig-Holstein, Campus Kiel, Kiel, Germany; 66 Institute for Molecular Medicine FIMM, University of Helsinki and Public Health Genomics Unit, National Institute for Health and Welfare, Helsinki, Finland; 67 Department of Biostatistics, Center for Statistical Genetics, University of Michigan, Ann Arbor, Michigan 48109, USA; 68 Department of Twin Research and Genetic Epidemiology, King's College London, SE17EH London, United Kingdom; 69 Cardiothoracic Surgery Unit, Department of Molecular Medicine and Surgery, Karolinska Institutet; 70INSERM UMR_S 937 & ICAN Institute for Cardiometabolism and Nutrition, Pierre and Marie Curie University, Paris 6, France; 71Institute for Genomics and Multiscale Biology, Department of Genetics and Genomic Sciences , Mount Sinai School of Medicine, US; 72Department of Human Genetics, McGill University, Montréal, Canada; 73 Division of Research, Kaiser Permanente Northern California; 74 Department of Experimental immunohematology, Sanquin, Amsterdam, The Netherlands; 75Medical

university Center Mainz, Dept. Of Medicine II, Mainz, Germany; 76Department of Haematology, University of Cambridge, CB2 0PT UK; 77NHS Blood and Transplant, Cambridge, CB2 0PT, UK; 78University of Texas Health Science Center, Human Genetics Center, Houston, Texas, USA; 79Department of Biostatistics, Boston University School of Public Health, Boston & National Heart, Lung, and Blood Institute's Framingham Heart Study, Framingham, Massachusetts, USA; 80Department of Epidemiology, Erasmus Medical Center, Rotterdam, The Netherlands ; 81Icelandic Heart Association, Kopavogur, Iceland; 82Transplantation Laboratory, Haartman Institute, University of Helsinki, Helsinki, Finland; 83 Department of Medicine, Landspitali University Hospital, Reykjavik, Iceland.; 84INSERM UMRS872, Cordeliers Research Centre, Paris, France; 85 Department of Clinical Physiology, Tampere University Hospital and University of Tampere, Tampere 33521, Finland; 86Unit for Molecular Epidemiology, Helmholtz Zentrum München - German Research Center for Environmental Health, Neuherberg, Germany; 87 Institute of Epidemiology II, Helmholtz Zentrum München - German Research Center for Environmental Health, Neuherberg, Germany; 88Department of Public Health & Clinical Medicine, Section for Nutritional Research, Umeå University, Umeå, Sweden; 89Cardiology Division, Department of Internal Medicine, Cardiovascular Genome Center, Yonsei University; 90Department of Internal Medicine, Cardiovascular Center, Seoul National University Hospital, Seoul , Korea; 91 Division of Cardiology, Samsung Medical Center, Seoul, Korea; 92 Science Center, Tampere University Hospital, Finland; 93Department of Medicine, University of Eastern Finland and Kuopio University Hospital, Kuopio, Finland; 94Center for Human Genetic Research, Massachusetts General Hospital and Harvard Medical, Boston, USA; 95Institut Pasteur de Lille, INSERM U744, Université Lille Nord de France, F-59000 Lille, France; 96Department of Epidemiology and Public Health, EA3430, University of Strasbourg, Strasbourg, France; 97Department of Cardiology, Toulouse University School of Medicine, Rangueil Hospital, Toulouse, France; 98Munich Heart Alliance, Munich, Germany; 99Department of Experimental Medicine, University of Milano-Bicocca, Monza, Italy; 100EPIMED Research Centre, Department of Clinical and Experimental Medicine, University of Insubria, Varese, Italy; 101Centre for Public Health, The Queen's University of Belfast, Belfast, Northern Ireland; 102UKCRC Centre of Excellence for Public Health (NI), Queens University of Belfast, Northern Ireland; 103 Lerner Research Institute, Cleveland Clinic, Cleveland, Ohio, USA; 104 Duke University; 105Cardiovascular Research Institute, Washington Hospital Center, 110 Irving St NW, Washington, DC 20010, USA; 106Division of Translational Medicine and Human Genetics, Department of Medicine, Perelman School of Medicine at the University of Pennsylvania, Philadelphia, PA 19130, USA; 107Department of Molecular Biology and Center for Human Genetic Research, Massachusetts General Hospital, Boston, USA; 108Department of Genetics, Harvard Medical School, Boston, USA; 109Biotherapeutics and Bioinnovation Center, Pfizer Inc, USA; 1101st Cardiology Department, Onassis Cardiac Surgery Center 356, Sygrou Ave, Athens, Greece; 111Department of Internal Medicine II - Cardiology, Ulm University Medical Center, Ulm, Germany; 112Division of Cardiovascular and Diabetes Research, Multidisciplinary Cardiovascular Research Centre, Leeds Institute of Genetics, Health and Therapeutics, University of Leeds, Leeds, UK; 113 Division of Epidemiology, MCRC & Leeds Institute of Genetics, Health and Therapeutics, University of Leeds, Leeds, UK; 114 Division of

Cardiovascular and Neuronal Remodelling, Multidisciplinary Cardiovascular Research Centre, Leeds Institute of Genetics, Health and Therapeutics, University of Leeds, UK
